# Exposures and suspected intoxications during SARS-CoV-2 pandemic: preliminary results from an Italian poison control centre

**DOI:** 10.1007/s11739-021-02774-0

**Published:** 2021-06-06

**Authors:** Giada Crescioli, Cecilia Lanzi, Francesco Gambassi, Alessandra Ieri, Anita Ercolini, Giulia Borgioli, Alessandra Bettiol, Alfredo Vannacci, Guido Mannaioni, Niccolò Lombardi

**Affiliations:** 1 Department of Neurosciences, Psychology, Drug Research and Child Health, Section of Pharmacology and Toxicology, University of Florence, Viale G. Pieraccini, 6-50139 Florence, Italy; 2 Tuscan Regional Centre of Pharmacovigilance, Florence, Italy; 3 Toxicology Unit and Poison Center, Careggi University Hospital, Florence, Italy; 4 Department of Experimental and Clinical Medicine, University of Florence, Firenze, Italy

**Keywords:** SARS-CoV-2, Pandemic, Exposure, Intoxication, Clinical toxicology, Poison control centre

## Abstract

Data on cleaner and disinfectant exposure and misuse-related acute intoxications in Italy during SARS-CoV-2 pandemic are still lacking. The aim of the present study was to analyse and describe cleaner and disinfectant-related intoxications during SARS-CoV-2 pandemic in an Italian poison control centre. Data were obtained from the toxicological consultations requested to the Toxicology Unit and Poison Centre, Careggi University Hospital, Florence (Italy). We compared data from January 1st to April 30th of 2019 and 2020. Data concerning probable or acute intoxication from any causative agent in the general population (all age groups), from private individuals or from Regional and National health structures, were included in the analysis. A toxicological evaluation was also performed to calculate the Poisoning Severity Score.

In 2019, 451 phone counselling sessions were performed and compared to a total of 410 calls received during the same period of 2020. In both periods, the majority of events occurred in paediatric (0–17 years) and adult (18–65 years) patients, who were mainly exposed to one toxic agent, and intoxications took place principally at home due to domestic accidents. The oral route of intoxication was the most frequently observed one, followed by inhalation of toxic agents, which increased by 4.7% in 2020. In 2020, sanitizers and cleaners were reported in 21.6% of cases compared to 12.5% in 2019. This is the first study describing cleaner and disinfectant-related intoxications in Italy. Our results suggested a possible misuse of these products during the SARS-CoV-2 pandemic, underling the effects of home isolation on mental health and unintentional toxic exposures.

## Introduction

Among precautions recommended to prevent SARS-CoV-2 transmission, besides wearing a mask, the proper cleaning and disinfection of hands and high-touch surfaces is of utmost importance [1]. Therefore, during the COVID-19 pandemic, hand sanitizers, detergents and disinfectants became fundamental necessities worldwide, and their supply was quickly depleted [2]. The World Health Organization (WHO) proposed a formula to easily make hand sanitizers at home [3], and several indications have been spread about surface cleaning according to scientific results on SARS-CoV-2 surviving in the environment. In fact, as for severe acute respiratory syndrome (SARS) and Middle East respiratory syndrome (MERS) viruses, SARS-CoV-2 may be transmitted both through droplets and hand self-delivery to mouth, eyes or nose after the contact of an infected surface [4]. Against coronaviruses, alcohol with 70–90% concentration, quaternary ammonium compounds, and hydrogen peroxide resulted as effective as antimicrobials [5]. Hypochlorite-based products in a concentration of 0.1% (1000 ppm) should be used after a proper cleaning of surfaces with soap and water or detergent to avoid hypochlorite degradation by organic material, while application of spray disinfectants is not recommended [6]. Information was rapidly disseminated worldwide, and in parallel with those coming from institutional sources, lots of people tried several “do-it-yourself” solutions to produce hand sanitizer [2] or they mixed detergents for house cleaning. Misuse of disinfectants and cleaning products, especially in closed environments, such as homes, could lead to unintentional intoxication both in adults and children [7]. In particular, results coming from a recent survey on USA adults, revealed gaps in knowledge on safe preparation of cleaning and disinfectant solutions, use of protective equipment when using cleaners and disinfectants, and safe storage of such products. Participants also reported washing food with bleach, applying household cleaning or disinfectant to bare skin, and intentionally inhaling or ingesting hand sanitizer or cleaner solutions [8]. Adverse effects related to the misuse of cleaning products and sanitizers include irritation of high airways or breathing problems, skin or eyes lesions, dizziness, headache, nausea and vomiting, and fatalities have also been reported [7, 9, 10]. A significant increase of approximately 20% in calls to poison control centres (PCCs) was observed throughout the USA in the first quarter of 2020 compared to the same period of 2019 [11]. The majority of calls concerned bleach exposure, and hand sanitizer/non-alcohol disinfectant misuse-related intoxications.

Italy was the first European country to experience the SARS-CoV-2 pandemic outbreak and the first one to cope with this new disease [12]. Nevertheless, data on exposures and suspected intoxications in Italy are still lacking, particularly those related to hand sanitizer, hand cleaner and detergents. In this context, the aim of the present study was to analyse and describe exposures and suspected intoxications during the SARS-CoV-2 pandemic from an Italian PCC.

## Methods

This study was performed on data obtained during phone counselling sessions by the Toxicology Unit and PCC, Careggi University Hospital, Florence (Italy). We compared data obtained from January 1st to April 30th of 2019 and 2020.

Data concerning exposures and probable or acute intoxications from any causative agent in adults and children (all age groups), from private individuals or from Regional and National health structures, were included in the analysis. For each phone counselling (first contact), after obtaining the patients’ informed consent to participate in the study, trained clinical toxicologists collected the following information: (1) patient’s demographic characteristics (age, gender); (2) number of suspected toxic agents and their description; (3) the place of intoxication occurrence (i.e. home, public closed places, workplace, open environment/outdoors); (4) description of the event and its circumstances (i.e. domestic accidents, voluntary intoxication, environmental conditions); (5) intoxication route. Toxic agents were categorised as follows: sanitizer/cleaners; acids/caustic sodas; bleaches; machine detergents; chloride vapours; hand washing detergents; thermometers fluids; other home cleaning products; fertilisers; glues; silica gel; paints/varnish; batteries; others toxic agents.

A toxicological evaluation was then performed to calculate the Poisoning Severity Score (PSS) [13] (PSS score 0—NONE: no signs or symptoms related to exposure; PSS score 1—MINOR: mild, transient symptoms with spontaneous resolution; PSS score 2—MODERATE: evident or prolonged symptoms; PSS score 3—SEVERE: severe or life-threatening symptoms; PSS score 4—FATAL: death), and to define the plausibility of the intoxication. Intoxications were defined as absent (no intoxication), doubtful, possible, and confirmed. Toxicologists could also judge patients’ symptoms as “independent from the intoxication”. Moreover, data on toxicologists’ advice (i.e. observation at home, emergency department visit, follow-up by the general practitioner, and hospitalisation) and prescriptions, such as symptomatic/antidotal therapies, elimination of the toxic agents/products, were also recorded. After the first phone call counselling, all patients were re-contacted for a clinical and toxicological follow-up.

Data were described as number and percentages and compared between the two periods of observation (January 1st to April 30th, 2019 and January 1st to April 30th, 2020) with the Chi-square test. Statistical significance was considered with a *p* value ≤ 0.05. Statistical analyses were carried out using STATA-16.

## Results

Between January 1st and April 30th, 2019 the Toxicology Unit and PCC of Florence (Italy) received a total of 451 counselling calls, compared to a total of 410 calls received during the same period of 2020. The majority of events occurred in paediatric (0–17 years) and adult (18–65 years) patients during both observational periods (93.2% and 86.6% in 2019 and 2020, respectively), and no statistically significant differences were observed between the two periods regarding patients’ gender, number of toxic products, the place where the intoxication occurred and related circumstances. Both in 2019 and 2020, the majority of subjects were exposed to one toxic agent (98.7% and 97.6%, respectively) and intoxications took place mainly at home (92.0% and 93.2%) due to domestic accidents (95.1% and 94.4%). Voluntary intoxications accounted for only 3.5% in 2019 and 5.4% in 2020 (Table 1).

**Table 1:** Patients’ characteristics

	Jan–Apr 2019	Jan–Apr 2020	*p* value
	Tot 451	Tot 410	
	*N* (%)	*N* (%)	
Age classes (years)			
Paediatrics (0–17)	207 (45.90)	177 (43.2)	0.235
Adults (18–65)	200 (44.34)	178 (43.4)	
Elderly (> 65)	44 (9.76)	55 (13.4)	
Gender			
M	213 (47.2)	203 (49.5)	0.261
F	221 (49.0)	199 (48.5)	
Not reported	17 (3.8)	8 (2.0)	
Number of toxic agents			
1	445 (98.67)	400 (97.56)	0.229
More than 1	6 (1.33)	10 (2.44)	
Place (where)			
Home	415 (92.02)	382 (93.17)	0.167
Public closed places	16 (3.55)	12 (2.93)	
Workplace	17 (3.77)	9 (2.20)	
Outdoors	3 (0.67)	3 (0.73)	
Others	–	4 (0.98)	
Circumstances (why)			
Domestic accidents	429 (95.12)	387 (94.39)	0.174
Voluntary intoxication	13 (3.55)	22 (5.37)	
Environmental condition	2 (0.44)	–	
Others	4 (0.89)	1 (0.24)	

Through the first phone counselling and the follow-up call, the clinical and toxicological evaluations performed by trained clinical toxicologists assigned a PSS score of 0 to the majority of cases of probable or acute intoxications in both periods of observation (52.5% vs. 60.7%). No fatal cases were observed neither in 2019, nor in 2020. The oral route of intoxication was the most frequently observed one (83.8% vs. 79.3%), followed by inhalation of toxic agents, which increased by 4.7% in 2020. Intoxication was judged as “absent” or “doubtful” in almost 80% of cases both in 2019 and 2020, and “confirmed” only in 9.3% of cases in 2019 and 10.5% in 2020. Overall, symptoms reported by patients during phone counselling were judged as “independent from the intoxication” in 10 cases, 6 cases (1.3%) in 2019 and 4 cases (1%) in 2020. Overall, 38.6% of patients in 2019 and 40.2% in 2020 required prescription of a targeted therapy. In the majority of cases, therapies were symptomatic (36.4% vs. 37.8%), and intoxications improved completely in all cases herein analysed (Table 2). Considering both periods, antidotes were prescribed to five patients, but none of these related to poisoning from detergents and disinfectants. In 2019, two patients were exposed at home to antifreeze and to ethylene glycol, respectively. They were treated at home with alcohol as specific detoxifying agent and counselled to visit the emergency department in case of worsening. In 2020, one patient was accidentally exposed to ethylene glycol. He was admitted to the emergency department without apparent symptoms of intoxication, and prescribed intravenous ethanol administration. Another patient was hospitalised due to accidental exposure to hydrofluoric acid. He was administered calcium gluconate as a specific antidote. The last patient reported a burn while at work and was successfully treated at the emergency department. The burn was caused by a specific detergent for use in the laboratory mixed with hot water.

**Table 2:** Toxicological evaluation

	Jan–Apr 2019	Jan–Apr 2020	*p* value
	Tot 451	Tot 410	
	*N* (%)	*N* (%)	
PSS—poisoning severity score			
PSS 0—none	237 (52.55)	249 (60.73)	0.017
PSS 1—minor	168 (37.25)	126 (30.73)	
PSS 2—moderate	26 (5.76)	24 (5.85)	
PSS 3—severe	3 (0.67)	6 (1.46)	
PSS 4—fatal	–	–	
Not assessed	17 (3.77)	5 (1.22)	
Route of intoxication			
Oral	378 (83.81)	325 (79.27)	0.229
Inhalation	47 (10.42)	62 (15.12)	
Ocular	14 (3.10)	12 (2.93)	
Cutaneous	12 (2.66)	11 (2.68)	
Intoxication plausibility			
Absent	231 (51.22)	213 (51.95)	0.603
Doubtful	120 (26.61)	91 (22.20)	
Possible intoxication	51 (11.31)	58 (14.15)	
Confirmed intoxication	42 (9.31)	43 (10.49)	
Symptoms independent from intoxication	6 (1.33)	4 (0.98)	
Not assessed	1 (0.22)	1 (0.24)	
Toxicologist advices			
Observation at home	197 (67.01)	293 (72.35)	< 0.001
Emergency department visit	27 (9.18)	64 (15.80)	
Follow-up by general practitioner	35 (11.90)	27 (6.67)	
Hospitalisation	35 (11.90)	21 (5.19)	
Prescribed therapies			
No	277 (61.42)	245 (59.76)	0.968
Yes			
Symptomatic therapies	164 (36.36)	155 (37.80)	
Elimination of toxic substances	6 (1.33)	5 (1.22)	
Antidotal therapies	2 (0.44)	3 (0.76)	
Other	2 (0.44)	2 (0.49)	

Reporting of sanitizer/cleaners, bleaches, chloride vapours, fertilisers, glues, silica gel, paints/varnish, and batteries as intoxication causative agents increased in the first quarter of 2020, compared to the same period of 2019. In particular, in 2020, sanitizer/cleaners, bleaches and chloride vapours were reported in 21.6%, 14.4%, and 6.6% of cases, respectively, compared to 12.5%, 10.7%, and 3.3% in 2019 (Table 3).

**Table 3:** Products involved in poisoning

Agents	Jan–Apr 2019	Jan–Apr 2020	*p* value
	Total calls 451	Total calls 410	
	*N* (%)	*N* (%)	
Sanitizer/cleaners	57 (12.50)	91 (21.56)	< 0.001
Acids/caustic sodas	64 (14.04)	53 (12.56)	
Bleaches	49 (10.75)	61 (14.45)	
Machine detergents	35 (7.68)	33 (7.82)	
Chloride vapours	15 (3.29)	28 (6.64)	
Hand washing detergents	21 (4.61)	17 (4.03)	
Thermometers fluids	20 (4.39)	11 (2.61)	
Other home cleaning products	16 (3.51)	12 (2.84)	
Fertilisers	7 (1.54)	11 (2.61)	
Glues	7 (1.54)	8 (1.90)	
Silica gel	6 (1.32)	9 (2.13)	
Paints/varnish	5 (1.10)	10 (2.37)	
Batteries	4 (0.88)	9 (2.13)	
Others	150 (32.89)	69 (16.35)	

## Discussion

This study aimed to analyse and compare data collected from January 1st to April 30th of 2019 and 2020 during phone counselling by the Florence PCC. This analysis provided a useful picture of exposures and suspected intoxications during the SARS-CoV-2 pandemic. Compared to a small decrease in the total number of calls to the PCC in 2020, we observed an increase in calls concerning sanitizers/cleaners and detergents, bleaches, and chloride vapours as causative agents of intoxication. Even from a smaller sample, these results are in line with those already published in international literature [7, 9, 10]. During the National lockdown (March and April, 2020), Italian people were forced to stay at home. Thus, the exposure to these products become more frequent. The increase in reporting of chloride vapours as causative agent of intoxication and of inhalation as route of intoxication also suggests a possible in-home inappropriate use of sanitizer/cleaners (i.e. bleach and alcohol), that could be very dangerous if mixed. Moreover, the increase in reporting fertilisers, glues, and paints/varnishes suggest that these intoxications were related to the few domestic activities allowed during the lockdown, such as craft projects and house renovation.

Few studies have been published in the literature on this topic, most of which were performed on small population samples and with follow-up periods of variable duration. Fayed and Sharif described the pattern of toxic exposure among the cases referred to Tanta Poison Control Center (Tanta, Egypt) during 2016–2020, exploring the impact of lockdown due to the COVID-19 pandemic on the pattern of cases managed in the last year [14]. During the spring months (from March to May) of 2016–2020, a total of 1,916 patients with complete medical records were recruited. From their 5-year retrospective, comparative cross-sectional study, authors reported that there were delays in time from toxic exposure to emergency services during the lockdown period (from March to May 2020). This was reflected in significant lower recovery rates and higher death rates despite the marked decrease in the total number of hospital admissions in comparison to the past 4 years (2020 versus 2016–2019). Moreover, the lockdown period showed significantly higher phosphides and antipsychotics exposure than the previous years. However, predominance of female exposure and intentional self-poisoning was maintained over the past 5 years, including the lockdown.

For the 3-month period from January 1 to March 30, 2020, the number of calls about exposure to cleaners and disinfectants made to poison centres in all states of USA increased 20.4%, and the number of calls about exposure to disinfectants increased 16.4% [10]. In this context, Rosenman and Colleagues examined calls about cleaners and disinfectants to the Michigan Poison Center (East Lansing, United States) since the onset of the COVID-19 pandemic. The authors compared all calls related to exposure to cleaners or disinfectants, calls with symptoms, and calls in which a health care provider was seen during the first quarters of 2019 and 2020 and in relationship to key COVID-19 dates [15]. From 2019 to 2020, the number of all disinfectants-related calls increased by 42.8%, the number of calls with symptoms increased by 57.3%, the average number of calls per day doubled after the first Michigan COVID-19 case, from 4.8 to 9.0, and the proportion of calls about disinfectants among all exposure calls to the Michigan Poison Center increased from 3.5 to 5.0%. However, calls for exposure to cleaners did not increase significantly. Similarly to our results, exposure occurred at home for the majority of calls, and oral ingestion was the exposure route for over half of calls.

In addition, Yasseen Iii and colleagues analysed data from January to June in 2019 and 2020 from five Canadian poison centres, describing calls regarding selected cleaning products and presenting year-over-year percentage change [16]. According to the authors, there were 3408 (42%) calls related to bleaches; 2015 (25%) to hand sanitizers; 1667 (21%) to disinfectants; 949 (12%) to chlorine gas; and 148 (2%) to chloramine gas. These findings are comparable with our results, particularly in terms of the type of product responsible for the suspected intoxication. Otherwise, they observed an increase in overall calls occurred in conjunction with the onset of COVID-19, with the largest increase occurring in March.

Since the onset of the COVID-19 pandemic, poison centre calls regarding exposures to cleaners, disinfectants, and hand sanitizers have increased as compared with prior years indicating a need to evaluate household safety precautions. In this scenario, Gharpure and Colleagues, during May 2020, conducted an opt-in Internet panel survey of 502 U.S. adults [17]. Survey items evaluated knowledge regarding use and storage of cleaners, disinfectants, and hand sanitizers; attitudes about household cleaning and disinfection; and safety precautions practised during the prior month. The authors assigned a knowledge score to each respondent to quantify knowledge of safety precautions and calculated median scores by demographic characteristics and attitudes, identifying relevant gaps in knowledge regarding safe use and storage of cleaners, disinfectants, and hand sanitizers. Knowledge scores were lower among younger than older age groups and among black non-Hispanic and Hispanic respondents compared with white non-Hispanic respondents. In their discussion, the authors stated that tailored communication strategies should be used to reach populations with lower knowledge of cleaning and disinfection safety. In addition, they affirmed that as knowledge alone did not shape individual engagement in safety precautions, health promotion campaigns may specifically emphasise the health risks of unsafe use and storage of cleaners, disinfectants, and hand sanitizers to address risk perception.

No official data from Italy are currently available in medical literature to compare results of our evaluation. Only a press release from the PCC of Milan (Hospital NIGUARDA, Lombardy Region) claimed an increase of about 65% of counselling for household poisoning related to the use of disinfectants in the overall population. Percentages raised to 135% in case of accidental exposure of paediatric patients [18]. In our sample, during the same period, call counselling related to children, adolescents and adults did not show an increase, while those related to elderly showed a slight increase. To date, evidence regarding accidental poisoning in elderly is still lacking. A possible explanation could be related to policy measures for physical distancing which, in elderly, increased mental health problems, particularly emotional loneliness [19]. Social isolation and loneliness in elderly may predispose to cognitive decline, facilitating the onset of new neuropsychiatric symptoms such as delirium, agitation and apathy [20]. In the context of home confinement and social isolation, elderly who had to handle sanitizers/cleaners and detergents independently, without the presence of their caregivers, may have been exposed to a higher risk of involuntary intoxication.

In our sample, the observation of symptoms at home increased by about 5%. This evidence reflects the need to avoid hospital admissions in case of low or moderate symptoms. In fact, due to the strict recommendations concerning hospital access during the lockdown in Italy [21], to contain SARS-CoV-2 infections, people were invited to access the emergency departments and hospitals only in case of urgent need.

## Conclusions

To the best of our knowledge, this is the first study describing exposures and suspected intoxications from any causative agents during the first wave of SARS-CoV-2 pandemic in Italy. Although this study can be considered a preliminary analysis, our results suggested possible in-home inappropriate use of sanitizers/cleaners and detergents, and great attention should be paid to the risk associated with involuntary intoxication and to the need of correct information concerning the utilisation of these products.

In this context, an active and well-functioning service such as the PCC may help patients to manage their symptoms at home and reduce emergency department crowding.

Further, larger multicentre studies, involving more than one PCC, are certainly needed to better characterise such events in the general population, particularly during the pandemic that we are experiencing at the time of writing, and during possible future waves of COVID-19 or other emergencies of the same kind.

## References

[1] WHO Official Updates - Coronavirus disease (COVID-19) advice for the public. https://www.who.int/emergencies/diseases/novel-coronavirus-2019/advice-for-public. Last accessed: 01 June 2021

[2] Hakimi AA, Armstrong WB (2020). Hand Sanitizer in a Pandemic: Wrong Formulations in the Wrong Hands. J Emerg Med.

[3] Guide to Local Production: WHO-recommended Handrub Formulations. https://www.who.int/gpsc/5may/Guide_to_Local_Production.pdf. Last accessed: 01 June 2021

[4] Kanamori H, Weber DJ, Rutala WA (2020). The role of the healthcare surface environment in SARS-CoV-2 transmission and potential control measures. Clin Infect Dis.

[5] Otter JA, Donskey C, Yezli S (2016). Transmission of SARS and MERS coronaviruses and influenza virus in healthcare settings: the possible role of dry surface contamination. J Hosp Infect.

[6] Cleaning and disinfection of environmental surfaces in the context of COVID-19. https://www.who.int/publications/i/item/cleaning-and-disinfection-of-environmental-surfaces-inthe-context-of-covid-19. Last accessed: 01 June 2021

[7] Kuehn BM (2020). More than 1 in 3 US adults use disinfectants unsafely. JAMA.

[8] Gharpure R, Hunter CM, Schnall AH (2020). Knowledge and practices regarding safe household cleaning and disinfection for COVID-19 prevention—United States, May 2020. MMWR Morb Mortal Wkly Rep.

[9] Kuehn BM (2020). Hand sanitizer poisoning and deaths reported in 2 states. JAMA.

[10] Yip L, Bixler D, Brooks DE (2020). Serious adverse health events, including death, associated with ingesting alcohol-based hand sanitizers containing methanol—Arizona and New Mexico, May–June 2020. MMWR Morb Mortal Wkly Rep.

[11] Kuehn BM (2020). Spike in poison control calls related to disinfectant exposures. JAMA–J Am Med Assoc.

[12] Indolfi C, Spaccarotella C (2020). The Outbreak of COVID-19 in Italy Fighting the Pandemic. Journals Am Coll Cardiol: Case Rep.

[13] Persson HE, Sjöberg GK, Haines JA, De Garbino JP (1998). Poisoning severity score. Grading of acute poisoning. J Toxicol-Clin Toxicol.

[14] Fayed MM, Sharif AF (2021). Impact of lockdown due to COVID-19 on the modalities of intoxicated patients presenting to the emergency room. Prehosp Disaster Med.

[15] Rosenman KD, Reilly MJ, Wang L (2021). Calls to a state poison center concerning cleaners and disinfectants from the onset of the COVID-19 pandemic through April 2020. Public Health Rep.

[16] Yasseen A, Weiss D, Remer S (2020). Increases in exposure calls related to selected cleaners and disinfectants at the onset of the COVID-19 pandemic: data from Canadian poison centres. Heal Promot Chronic Dis Prev Canada.

[17] Gharpure R, Miller GF, Hunter CM (2020). Safe use and storage of cleaners, disinfectants, and hand sanitizers: knowledge, attitudes, and practices among U.S. adults during the COVID-19 pandemic, May 2020. Am J Trop Med Hyg.

[18] ASST Grande Ospedale Metropolitano Niguarda. News (24.03.2020). https://www.ospedaleniguarda.it/news. Last accessed: 01 June 2021

[19] van Tilburg TG, Steinmetz S, Stolte E (2020). Loneliness and mental health during the COVID-19 pandemic: a study among dutch older adults. Journals Gerontol Ser B.

[20] Manca R, De Marco M, Venneri A (2020). The impact of COVID-19 infection and enforced prolonged social isolation on neuropsychiatric symptoms in older adults with and without dementia: a review. Front Psychiatry..

[21] Governo Italiano. Presidenza del Consiglio dei Ministri. Coronavirus, la normativa vigente (Decreto-legge 25 marzo 2020, n. 19). https://www.governo.it/it/coronavirus-normativa. Last accessed: 01 June 2021

